# Osteopathic Principles: The Inspiration of Every Science Is Its Change

**DOI:** 10.7759/cureus.12478

**Published:** 2021-01-04

**Authors:** Bruno Bordoni, Allan R Escher

**Affiliations:** 1 Physical Medicine and Rehabilitation, Foundation Don Carlo Gnocchi, Milan, ITA; 2 Anesthesiology/Pain Medicine, H Lee Moffitt Cancer Center and Research Institute, Tampa, USA

**Keywords:** osteopathic, fascia, touch, osteopathy, manual therapy

## Abstract

The Educational Council on Osteopathic Principles (ECOP) annually renews and reviews the fundamental osteopathic principles that Dr. Still left behind for osteopathic medicine (OM). These tenets represent a guide and rationale for the osteopathic manual approach. The non-profit research organization, Foundation of Osteopathic Research and Clinical Endorsement (FORCE), which was founded in 2013 under the auspices of different international professionals, wishes to propose changes to these principles based on scientific knowledge, which did not exist in the nineteenth century, as well as all the information discovered subsequently. The proposal is not a constraint, but a further stimulus to improve the vision of OM. We believe, in fact, that a principle or a point of view never ceases to evolve: the inspiration of every science is its change.

## Introduction and background

The osteopathic manual approach and osteopathic medicine (OM) were conceived and built from the studies and experience in the nineteenth-century of Andrew Taylor Still, DO [1]. According to Dr. Still, "Osteopathy, or osteopathic medicine, is a philosophy, a science, and an art" [1]. The foundations of OM emphasize the fact that osteopathy should not be associated, from an intellectual and methodological point of view, as a branch of medicine, the latter being the dispensation of drugs and surgery; OM is a complete and independent system of care [2]. OM works in conjunction and cooperation with medical disciplines [2]. One of the top representatives of OM, Littlejohn JIM DO, Ph.D., stated that the latter is a growing discipline and its path is both innovation and scientific progression; each osteopath can develop their skills by deepening all other fields of medicine and science [2]. Dr. Still devised fundamental concepts for OM, which have always been updated and revised through various organizations, such as in 2013 with the Osteopathic International Alliance (OIA), and then constantly, with the Educational Council on Osteopathic Principles (ECOP) established by the American Association of Colleges of Osteopathic Medicine (AACOM), with the aim of revising and/or maintaining the concepts of the science and philosophy of osteopathic medicine [1]. ECOP draws up and updates the glossary of osteopathic terminology (since 1981) where the most salient osteopathic principles can be found: the human being is a dynamic unit of function; the body posses self-regulatory mechanisms that are self-healing in nature; structure and function are interrelated at all levels; rational treatment is based on these principles [1]. Before being recognized as valid and accepted by the scientific community, osteopathic principles and OM have been subjected to multiple criticisms and the precursors of osteopathy have fought to see their beliefs recognized [3-5]. Many medical modalities at the time of their appearance, and very often, were opposed for different reasons (economic and personal interests, ignorance, presumption), as history teaches [6-8]. Making thought grow is always a source of effort and patience. Our non-profit research organization, Foundation of Osteopathic Research and Clinical Endorsement (FORCE), which was founded in 2013 under the aegis of diverse international professionals, has published many innovative articles in the field of manual medicine, clashing with habits and thought patterns not always easy to overcome for different reasons [9-12]. The intent of the article is to review some osteopathic principles, in particular, the second and third, trying to contribute to the development of OM based on different sciences and innovative points of view. We believe, in fact, that a principle or a point of view never ceases to evolve: the inspiration of a science is its change.

## Review

The human being is a dynamic unit of function

According to Dr. Littlejohn, OM is not a discipline that looks at the body only from one point of view but integrates all scientific knowledge with the ultimate goal of observing the body system as a whole [2]. A science that should be integrated into the osteopath's gaze is quantum physics, as its assumptions and mathematical demonstrations highlight many concepts that go beyond physics alone. The fine structure constant (whose mathematical resultant is α-1 = 137.035999070 (98) [0.71 parts on a billion]) is a dimensionless constant, which allows us to understand that the presupposition of a given science or reasoning is always bound by who proposes it and by who interprets it, as well as the final result [13]. This anthropic interpretation highlights the fact that the point of arrival is like the horizon, that is, it is not really reached, but there is the illusion of reaching it; to think that a principle is absolute is a mathematical, physical, and medical error. It is, therefore, legitimate to try to improve OM thinking. The unity of the human body emphasizes the fact that each body part is in communion; a body area could be affected by a local or distant dysfunction [14]. A visionary of OM was Irvin Korr, who stated, referring to the concept of bodily unity: "The person is the environment in which parts exist and operate" [14]. Additionally, he wrote that a person's life and the way of living and experiencing life are factors capable of influencing every single cell, to the point of involving the entire body system. To understand man, from a scientific point of view, we need to study man [14]. The osteopath could take a step forward. According to quantum physics, time and space do not exist and each particle is connected to another, whose bond becomes indissoluble once contact is created [15-17]. The communion of each molecule that makes up the body and the molecules outside our body can create a phenomenon known as entanglement. It is known that quantum entanglement arises when the wave function of a particle system cannot be represented as a product of the wave functions of each particle. It is the inseparable union of all molecules in a perpetual pleiotropic still image and in a harmonious entropy. Cell memory always exists and is collected by the Duan-Lukin-Cirac-Zoller scheme [18-19]. Each mechanical alteration of the cells creates a vibration or electromagnetic phenomenon, which emits quanta of light or biophotons (for the body and outside the body) and sounds or biophones (quanta of vibrational energy) [20-21]. The body receives and emits biophotons and biophones from the environment and for the environment in which it resides, respectively. This quantum information influences the electron rotation, polarization, and depolarization of cell membranes [22-23]. Depending on the mode of movement of the electrons, quantum information (light and sound) is generated, which can form memory "files" (Qutrit); it is the imprint of what we are and what we receive [24]. We are an electromagnetic coherence that makes our consciousness a form (body) and intention (movement/non-movement). We are the result of multiple electromagnetic vibrations, which interface with other electromagnetic waves. There is no neutrality, but balance (in health); there is no immobility, but constant motions of different electromagnetic spectra. There is not even silence because everything emits sound. The osteopathic principle of body unity could be defined in another way, according to FORCE: the body is in the whole; the body continuum merges with everyone's body continuum and penetrates the environment in which one lives. This concept also comes together to understand the contact modality between osteopath and patient, that is, palpation and therapeutic intention and the concept of osteopathic dysfunction. We should imagine ourselves in a matrix in which we are immersed, and we constitute its essence. Health has an oscillatory organization different from disease; the matrix can influence our state and vice versa [25-28]. In the same way, our movement, therefore the will and the thought to act, directs our oscillatory three-dimensionality in the matrix. The intention to change dysfunction in health means imposing our infinite oscillations on others [29]. It must be understood that all oscillations are in contact with everything that exists. Wanting to improve the patient's health status means wanting to improve our health and our surroundings. Dr. Littlejohn JM recalls that Hippocrates himself defined the disease as a disruption of the harmony of the body, its fluids, and its strength and that there are inherent vital bodily forces capable of allowing the patient's self-healing [2]. The osteopath's hand should not seek neutrality or stillness or silence; the hand should look for functional entropy. The latter is the organism's maximum adaptability that is expressed in health. On the contrary, the presence of dysfunctional syntropy or pattern implies the presence of a predominant pattern that breaks the balance [30]. We are already in deep contact with another individual (with everything and everyone) before touching a tissue. Deep palpatory or non-palpatory listening is simply accepting the presence of other individuals within us. The exchange is continuous and very fast. The hand emits electromagnetic frequencies, which oscillations turn into signals (electrical, biochemical). The hand is not just a touch, but a quantum instrument that interacts with multiple chaotic realities. One key for multiple locks. Our touch, our intention can enter the memory of the cells and create new memory. The most effective osteopathy is the thinking hand. Religion and science show us that there are no physical boundaries and we are one [31-33]. Our therapeutic imprint is already within the patient, just as the ability to understand the cause of the dysfunction is already inherent in our research. Aligning and collimating this already existing link is probably one of the foundations of the osteopathic approach. Quantum physics also confers reinforcement for the second osteopathic principle: the body posses self-regulatory mechanisms that are self-healing in nature. Electromagnetism and molecular vibrations induce a positive or negative adaptation to the person's health. Touch is felt from the epidermis to the deepest cells of the last tissue [34].

Structure and function are interrelated at all levels

The meaning of this assumption is that the shape of a structure influences the function of the same structure, just as the function equally influences the shape of the structure [1]. According to new information in the embryological and genetic fields, it is not the binomial form-function that is decisive for forming a salutogenic environment but, rather, the position. The phenotype (form and function) of a living organism derives from the genotype (genetic information); epigenetics will determine the modality and extent to which genes are activated [35]. Chromosome territories are nuclear spaces where chromosomes reside; their order and the way in which they are organized are fundamental to allow them to express their potential [36]. The spatial position of the deoxyribonucleic acid (DNA) constituents is the main requirement, without which the form-function of any biological organization could not exist [37]. Epigenetic modifications are the result of changes that go beyond the mere DNA sequence but depend on the methylation of histones (basic proteins that make up chromatin, for about 90%) and on the acetylation of DNA [38]. The N-terminal tail histones are able to undergo various modifications thanks to the presence of numerous enzymes influencing the transcription of a gene; the presence and, therefore, the position of a specific enzyme will create a specific epigenetic response in cascade [38]. The ability to create a form and a function is predetermined by the specific presence of these enzymatic bonds; it is the position that will determine the binomial form-function. The specific position of a DNA strand will determine a specific response to the behavior of that specific DNA [39]. It is shown that depending on the location of certain mesodermal cells, a specific area of the heart will be synthesized and not others, underlining that the study of embryology and genetics should be sciences taken into consideration to further understand osteopathic principles [40]. The structure-function is not the question but the answer. Cellular mechanotransduction occurs thanks to the change in the shape of the cell structures and with the displacement of fluids, both inside and outside the cell itself [41]. We know that in the moment of the passage of mechanical forces, not all cell structures respond in unison, but only thanks to the position of specific enzymes and ion channels or the position of certain proteins in a specific area [42]. What could manual osteopathic treatment do in consideration of these notions: look for the shape and function of a structure or create space between tissues? Space implies the ability to move and the possibility of different body components to regain the position before the dysfunction and to be able to express themselves at their best. Space is movement. It is from space that osteopathy begins because space is health. Space creates the possibility of optimal expression of all the specific structures of a given anatomic area. All research in the osteopathic field to restore function and/or form, respecting the limits of the dysfunction itself, have had positive results thanks to the creation of movement, which is allowed by space; space helps create those conditions for which tissues regain their ability to respond to specific mechano-metabolic signals. To give an example, an osteopathic approach to improve symptoms in the presence of gastroesophageal reflux does not mean having altered the structures or shapes of the gastric tract [43]. In this cited study, osteopathic physicians achieved positive results thanks to cervical mobilization. Acting on the possibility of giving movement means creating a sufficient space for which neurological afferents and efferences have obtained the ability to use their maximum capacity for expression. As already written in a previous article: “The main objective of the osteopath and that of osteopathic manipulative medicine (OMM) is to create space between the different tissues. The sliding capacity of the various tissue layers and between the different body components, up to the possibility of movement between cells is the salutogenic stimulus to allow the circulation of fluids, the biochemical exchange, and the adequate management of the multiple internal and external stimuli that perturb the body living. Movement is allowed by space and space is life" [44]. Space or position is used for movement and to give the possibility to all the structures in that anatomical area to be able to function, respecting the purpose with which they were positioned in that specific area. Our proposal to try to improve the previous osteopathic paradigm is: the position of a body structure decides its shape and function. The fourth osteopathic principle, "rational treatment is based on these principles" implies a reconsideration of the therapeutic path, involving new disciplines such as quantum physics, embryology, and genetics.

## Conclusions

The article discussed the possibility of reviewing and refining the two of the four osteopathic principles conceived by Dr. Still and subsequently renewed annually by the Educational Council on Osteopathic Principles (ECOP). According to the vision of our non-profit group, the FORCE, certain scientific disciplines should be given greater consideration to understand and possibly implement the osteopathic concepts mentioned above. Quantum physics, embryology, and genetics, that is, all the sciences that could allow us to observe the osteopathic approach with more points of view. We are convinced that the constant improvement and reformulation of some concepts, reflecting new scientific information, is the lifeblood of knowledge. To conclude, the osteopathic principles could be: the body is the whole; the body continuum merges with everyone's body continuum and penetrates the environment in which one lives; the body posses self-regulatory mechanisms that are self-healing in nature; the position of a body structure decides its form and function; rational treatment is based on these principles.
